# A Host-Specific Blocking Primer Combined with Optimal DNA Extraction Improves the Detection Capability of a Metabarcoding Protocol for Canine Vector-Borne Bacteria

**DOI:** 10.3390/pathogens9040258

**Published:** 2020-04-01

**Authors:** Lucas G. Huggins, Anson V. Koehler, Bettina Schunack, Tawin Inpankaew, Rebecca J. Traub

**Affiliations:** 1 Faculty of Veterinary and Agricultural Sciences, University of Melbourne, Parkville, Victoria 3050, Australia; anson.koehler@unimelb.edu.au (A.V.K.); rebecca.traub@unimelb.edu.au (R.J.T.); 2 Bayer Animal Health GmbH, 51373 Leverkusen, Germany; bettina.schunack@bayer.com; 3 Faculty of Veterinary Medicine, Kasetsart University, Bangkok 10900, Thailand; tawin.i@ku.th

**Keywords:** canine vector-borne disease, blocking primers, blood DNA extraction, next-generation sequencing, kit contaminant bacteria

## Abstract

Bacterial canine vector-borne diseases are responsible for some of the most life-threatening conditions of dogs in the tropics and are typically poorly researched with some presenting a zoonotic risk to cohabiting people. Next-generation sequencing based methodologies have been demonstrated to accurately characterise a diverse range of vector-borne bacteria in dogs, whilst also proving to be more sensitive than conventional PCR techniques. We report two improvements to a previously developed metabarcoding tool that increased the sensitivity and diversity of vector-borne bacteria detected from canine blood. Firstly, we developed and tested a canine-specific blocking primer that prevents cross-reactivity of bacterial primer amplification on abundant canine mitochondrial sequences. Use of our blocking primer increased the number of canine vector-borne infections detected (five more *Ehrlichia canis* and three more *Anaplasma platys* infections) and increased the diversity of bacterial sequences found. Secondly, the DNA extraction kit employed can have a significant effect on the bacterial community characterised. Therefore, we compared four different DNA extraction kits finding the Qiagen DNeasy Blood and Tissue Kit to be superior for detection of blood-borne bacteria, identifying nine more *A. platys*, two more *E. canis*, one more *Mycoplasma haemocanis* infection and more putative bacterial pathogens than the lowest performing kit.

## 1. Introduction

Despite recent developments in veterinary care and medicine, canine vector-borne diseases (CVBD) continue to inflict a large burden with regard to morbidity and mortality on dogs across the globe [1,2,3]. This is especially true in low-socioeconomic countries, that may have little available resources to invest into disease prevention programs [1,2,3]. In particular, countries spanning the tropics must affront an expansive range of CVBDs that comprise a leading cause of fatality in dogs [3,4,5,6]. Bacterial infections can be some of the deadliest CVBDs in such regions with pathogens such as *Ehrlichia canis*, the causative agent of canine monocytic ehrlichiosis (CME), being the biggest contributor to a raft of life-threatening conditions, including pancytopenia, fever, bleeding tendencies and immunosuppression [7,8,9]. Other disease-causing species include, *Anaplasma platys* which is a cause of recurrent thrombocytopenia in canines [10], haemotropic mycoplasmas that are associated with haemolytic syndrome [6] and *Bartonella* spp. that can produce severe endocarditis [11]. Rates of canine infection with such pathogenic agents can be very high, especially in tropical countries of Southeast Asia, where, for example 25.5% of Malaysian, 21.8% of Cambodian and 9.9% of Thai dogs have previously been found positive for *E. canis* by PCR [3,6,8]. Lower but nonetheless significant levels of infection by haemotropic mycoplasmas (3.7–12.8%) and *A. platys* (3.7–4.4%) have also been detected in these same countries [6,8].

Exploration and surveillance of CVBD is not only important from a veterinary perspective but also a public health standpoint, as several bacterial CVBD agents are zoonotic. For example, *Rickettsia felis* and *Rickettsia conorii*, when transmitted to humans, are the causative agents of flea-borne spotted fever and Mediterranean/Indian/Israeli tick typhus, respectively, with both having dogs as reservoir hosts [12,13]. In addition, dogs are considered sentinel hosts for the zoonotic pathogens *Borrelia burgdorferi* sensu lato, which causes Lyme disease and *Anaplasma phagocytophilum* responsible for the potentially lethal human granulocytic anaplasmosis [14,15,16,17]. 

Thorough characterisation and monitoring of CVBD is crucial to protect the health of dogs and humans, especially in regions of Asia where there has been little explorative research done into established, emerging and novel CVBDs [2,5]. To address these knowledge gaps, state-of-the-art methodologies could be employed such as next-generation sequencing (NGS) based 16S ribosomal RNA (16S rRNA) metabarcoding that can supersede conventional PCR (cPCR) techniques with regard to their ability to detect and discover rare and/or novel organisms [18,19]. 16S rRNA metabarcoding is better able to elucidate diversity in explorative research by not relying on likely pathogen prevalence in a region nor on *a priori* knowledge of a pathogen’s target genetic sequences, whilst also being better able to characterise coinfection [18,20]. Previous work has already developed a novel 16S and 18S rRNA metabarcoding methodology to characterise the range of blood-borne bacterial, apicomplexan and kinetoplastid organisms infecting canine hosts in Thailand, a country of substantial CVBD diversity [4,8,21,22,23]. The current research takes this further, by tackling some of the inherent challenges of NGS microbiome research whilst also improving methods to be better at unearthing pathogen diversity in the context of the canine blood micro-environment [24]. 

Prior research employing NGS metabarcoding analysis of bacterial pathogens from canines suffered from issues of bacterial primer cross-reactivity with the canine mitochondrial 12S ribosomal RNA (12S rRNA) gene [22]. On average, 47% of total reads during previous experiments were from mitochondrial DNA (mtDNA) cross-reactivity despite poor complementarity between the bacterial primers and 12S rRNA gene sequences [22]. This cross-reactivity is likely due to multiple causes, including a dominance of canine host DNA in the blood [22] in conjunction with the prokaryotic origin of mitochondria producing sequence similarities [25]. 

Similar challenges have been tackled before by using blocking primers that selectively bind to and prevent the amplification of DNA sequences that would otherwise dominate amplicons in contexts where there is a stacked ratio of low copy number DNA of interest compared to overabundant DNA that is ideally excluded [26,27,28,29,30,31]. Considering this, we designed and tested a 3′-spacer C3 blocking primer that could prevent amplification of the canine 12S rRNA gene by our previously tested bacterial-universal primers [22]. We then compared our bacterial NGS metabarcoding pipeline with and without this blocking primer to assess its blocking efficacy and elucidate whether it improved bacterial detection capability. 

In addition, for NGS-based research, the method of DNA extraction employed can play a critical role in the diversity and sensitivity of 16S rRNA detected, particularly in the context of low biomass samples, such as blood [32,33,34]. Commercially available DNA extraction kits differ widely in the physical and chemical systems used from which they produce PCR-ready DNA as well as in their required time and complexity [34,35]. Recent attention has been brought to automated DNA extraction systems that simplify the researcher’s workload but that may also accrue a cost with regard to DNA quantity and quality for downstream applications [35,36]. Another variable that must be considered when determining the most appropriate DNA extraction kit for 16S rRNA metabarcoding research is that bacteria frequently contaminate kits and reagents [32,37]. Numerous studies have now explored this phenomenon via the use of rigorous negative controls to highlight the prevalence of common kit contaminant bacteria, also demonstrating that levels of kit contamination and the types of contaminants found can vary between kits and even batches within kits [32,33,38].

Taking this into consideration, we tested four DNA extraction systems. Two were a typical spin-column based whole blood DNA extraction procedure and two were automated systems employing the Maxwell^®^ RSC 48 Instrument (Promega, Madison, WI, USA). Of the two automated procedures, one was for extraction of whole blood and the other for extraction from the blood’s Buffy Coat layer, which has been reported to increase detection sensitivity of certain vector-borne bacterial species that are typically difficult to detect [39,40,41]. Both automated kits use magnetic bead extraction and purification. Kit performance was assessed and compared based on DNA yield, sensitivity of pathogen detection and presence of contaminant DNA. 

## 2. Results

### 2.1. Blocking Primer Performance

Initial experimentation using cPCR analysis with gel electrophoresis assessed the ability of our blocking primer: *Canis*-mito-blk, to bind to canine 12S mitochondrial sequences when it did not have the 3′-spacer C3 modification, which was observable via production of a 137 bp product (data not shown). When the blocking primer had the 3′-spacer C3 added, this 137 bp product was no longer produced even when the bacterial 16S rRNA primers were present in the PCR, indicating complete blocking of canine 12S rRNA amplification. Bacterial primers were not prevented from amplifying bacterial sequences as an approximately 250 bp product was still produced by these primers when used on canine blood samples positive for the pathogens *E. canis*, *A. platys* and *R. felis* (data not shown). 

After bioinformatic processing and filtering of NGS data a total of 4,746,542 reads were accrued, a result within the typical range for amplicon-based Illumina sequencing at a high-quality Phred score of over thirty. From the total reads, 58% were from samples amplified without blocking primers and 42% from samples with blocking primers. 

**Figure 1:**
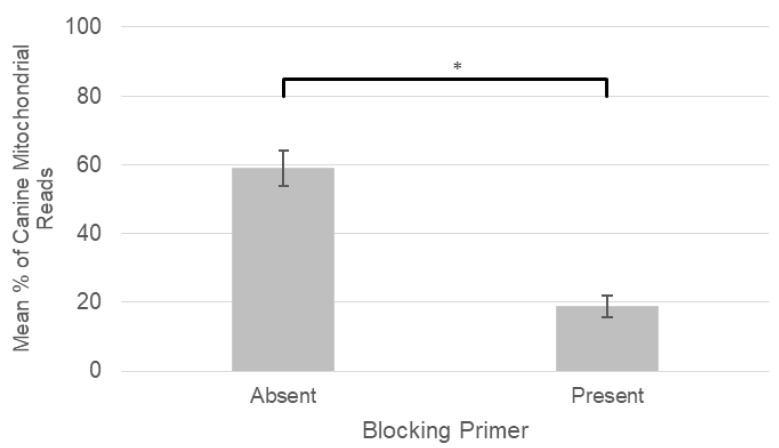
Mean percentage of canine mitochondrial reads across all samples in the absence and presence of the blocking primer. Vertical error bars display standard error, horizontal bar highlights a statistically significant difference at the *p* < 0.01 level (*).

Analysis of the mean percentage of canine mitochondrial reads relative to total reads on a per sample basis was 59% (±5.1%) for samples amplified without the blocking primer compared to 19% (±3.1%) with the primer (Figure 1). The difference between these two groups was statistically significant at the *p* < 0.01 level, indicating that our blocking primer significantly reduced the amount of canine mitochondrial reads amplified. Mean blocking efficiency across our canine DNA samples was 25% (±3.0%) according to the formula defined by Tan and Liu (2018) [27], with individual sample blocking efficiencies ranging from as low as 2.2% in some samples with low total sequence counts to as high as 100% blocking efficiency in others.

**Figure 2:**
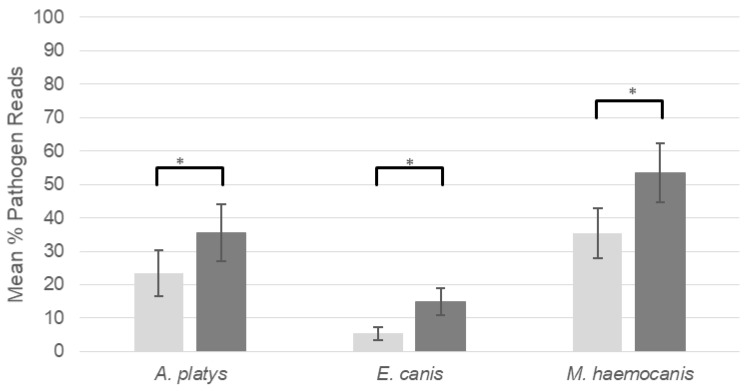
Mean percentage of three different pathogen reads across all pathogen positive samples in the absence of the blocking primer (light grey) and with the blocking primer (dark grey). Vertical error bars display standard error and horizontal bars highlight differences that are significant at the *p* < 0.01 level (*).

Pathogenic vector-borne bacterial species were detected from the species *E. canis*, *A. platys*, *M. haemocanis*, ‘*Candidatus* Mycoplasma haematoparvum’, *Mycoplasma turicensis*, *Bartonella* spp. as had been previously found by Huggins et al. (2019) [22]. However, only the high prevalence of *E. canis*, *A. platys* and *M. haemocanis*, permitted statistically comparable results between the ability of the metabarcoding methodology to detect vector-borne bacterial DNA with and without the use of *Canis*-mito-blk. When our blocking primer was used more *E. canis* and *A. platys* infections were detected by our bacterial metabarcoding method (Table 1). For *E. canis*, 24 infections were found using the blocking primer compared to 19 when they were not used, a similar increase in detection capability was found for *A. platys* with 20 infections detected using the blocking primer compared to 17 without. For detection of *M. haemocanis*, 19 infections were detected both with and without the blocking primer. The mean percentage of vector-borne bacterial reads with the blocking primer was always higher than when no blocking primers was used for; *E. canis* (15% ± 4.0% vs 5.4% ± 2.0%), *A. platys* (35% ± 8.5% vs 23% ± 7.0%) and *M. haemocanis* (53% ± 8.7% vs 35% ± 7.5%) with all differences being statistically significant (Figure 2). 

For samples with less common vector-borne bacteria, the percentage of pathogen reads compared to total reads was always higher for the samples that utilised the blocking primer. For example, for the two samples that were found to have a *Bartonella* spp. infection and two with a *Mycoplasma turicensis* infection, the average percentage of reads for the former pathogen was 1.5% vs 0.42% (with blocking primer vs without) and 16% vs 2.1% for the latter pathogen. A single sample with ‘*Candidatus* Mycoplasma haematoparvum’ reads had 54% of reads from this pathogen when using *Canis*-mito-blk, compared to 27% without. 

Comparing the number of bacterial taxonomic identifications across all samples with the blocking primer, it was found that there were 353 different sequences vs 327 without the blocking primer. Moreover, diversity indices calculated on just the bacterial portion of total sequences for all samples that used blocking primers vs all samples that did not, indicated that using the blocking primer improved characterisation of bacterial diversity. For both indices, a greater species diversity is demonstrated by a larger number. Using Simpson’s index, a value of 4.07 was acquired for samples using the blocking primer compared to 3.15 without (Table 2). With the Shannon–Wiener index, which takes into greater consideration rarer species with low read numbers, the index value was 2.3 when the blocking primer was used in contrast to 1.81 for samples without it [42] (Table 2). 

### 2.2. DNA Extraction Kit Performance

DNA extraction kit yield was compared which found that the automated extraction methods; Promega Buffy Coat (BC) = 680.2 ng/µL and Promega whole blood (WB) = 483.8 ng/µL obtained much higher average DNA yields than the non-automated kits Qiagen whole blood (QG) = 13.9 ng/µL and Bioline whole blood (BL) = 3.4 ng/µL (Table 3). At the same time, the range of DNA quantified in the extraction negative controls was also higher for the automated methods 0.005–0.024 ng/µL, while for QG, a lower range of 0.001–0.014 ng/µL was found and BL had a DNA yield below the lower limit at which the Qubit^TM^ can detect (Table 3). Such findings demonstrate a potentially higher initial quantity of bacteria or bacterial DNA in the automated extraction kits. 

Following bioinformatic processing, differing numbers of total raw and filtered reads were obtained for the datasets from each extraction method, an inevitable result of samples from each extraction method having to be run on separate NGS flow cells (Table 3). After filtering, the number of reads between datasets varied by a maximum of 1,157,220 reads. The total ASVs found across each kit’s dataset also differed greatly with QG kits finding the largest diversity of ASVs at 6683, followed by BL at 1401, WB at 435 and BC at 428 (Table 3). Counterintuitively, total reads post-filtering was not a good predictor of ASV diversity as QG, which had the lowest number of post-filtering reads and also had the highest ASV diversity. Additionally, BC had the highest post-filtering read total and the lowest ASV diversity. 

In a similar manner to the data from Thai dogs, the main vector-borne bacterial DNA found was from the species *E. canis*, *A. platys* and *M. haemocanis*, with differences in the numbers of infections elucidated between extraction kit types (Table 4). Across the 43 Cambodian blood samples compared, the QG kit found the highest number of infections for all three main pathogenic bacterial species at 35, followed by WB and BC with 28 and BL finding the least infections at 23 (Table 4). In addition, the QG kits found ASVs taxonomically classified as putative pathogens within the genera *Rickettsia* spp. and *Coxiella* spp., which were not detected by any of the other kit types. These taxonomic classifications could not be resolved to species level through either QIIME2 or a BLASTn search in GenBank due to very high sequence similarities between different species in the same genus. 

**Figure 3:**
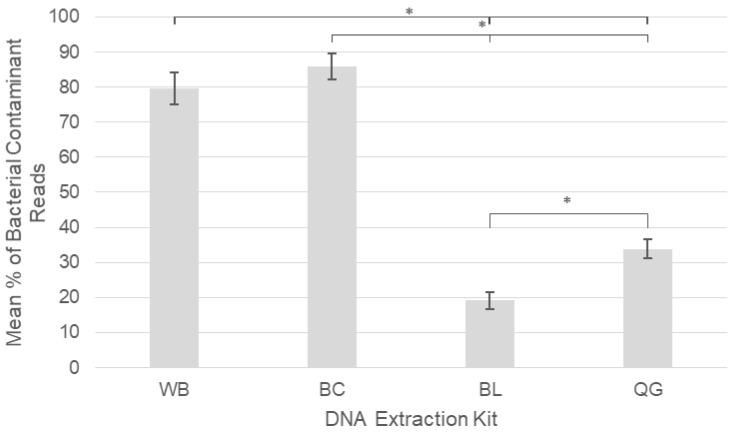
Mean percentages of kit-derived artificial bacterial contaminant reads relative to total reads across four different DNA extraction kits: Promega Whole Blood (WB), Promega Buffy Coat (BC), Bioline Whole Blood (BL), Qiagen Whole Blood (QG). Vertical bars display standard error and asterisks show differences between kit types that are significant at the *p* < 0.05 level (*).

The amount of artificial kit-derived contaminant DNA detected by our NGS methodology differed significantly depending on the extraction kit used (Figure 3). Both automated extraction kits had a significantly higher percentage of bacterial contaminant DNA (BC 86% and WB 80% of total reads) compared to 34% of reads for QG and 19% for BL. All differences between kits were significant at the *p* < 0.05 level apart from the mean contaminant reads between the two automated kits; WB and BC. 

The number of different ASVs assessed as being artificial kit contaminants due to them appearing with a read count of ≥100 in DNA extraction negative controls also varied with WB and BC kits having just four kit-contaminant ASVs, BL (n = 18) and QG (n = 57). Furthermore, the WB and BC kits demonstrated a distinct bacterial contaminant ‘fingerprint’, (Supplementary Table S1). For example, with the WB kits, results, on average, had a total of 51% of reads classified as *Massilia* spp. and 28% of reads as *Pseudomonas* spp. whilst BC kits on average had a composition of 61% *Massilia* spp. and 24% *Cellulomonas* spp. reads. In comparison, the composition of contaminant bacterial reads was less marked for the non-automated kits with QG having 7.9% of sequences classified as belonging to chloroplast DNA and 4.3% as *Burkholderia* spp., whilst BL kits had 4.8% of total reads on average belonging to *Burkholderia* spp. (Supplementary Table S1).

**Figure 4:**
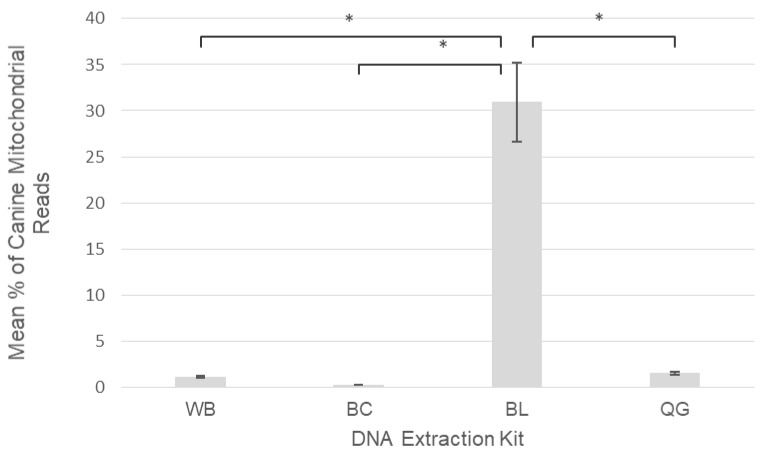
Mean percentages of canine mitochondrial reads relative to total reads across four different DNA extraction kits: Promega Whole Blood (WB), Promega Buffy Coat (BC), Bioline Whole Blood (BL), Qiagen Whole Blood (QG). Vertical bars display standard error and asterisks show differences between kit types that are significant at the *p* < 0.05 level (*).

The amount of cross-reactive amplification between the bacterial primers on canine mitochondrial sequences also differed significantly, depending on the DNA extraction kit used, despite blocking primers being present in all reactions. When samples were extracted using BL kits a statistically significant increase (*p* < 0.05) in the mean percentage of canine mitochondrial reads relative to total reads was observed with an average of 31% of reads being host mitochondrial sequences (Figure 4). This contrasted with much lower percentages of host mitochondrial sequences when samples were extracted with QG (1.5%), WB (1.1%) and BC (0.2%). 

## 3. Discussion

### 3.1. Blocking Primer Performance

The use of our blocking primer (*Canis*-mito-blk) was demonstrated to significantly reduce the amount of bacterial primer cross-reactivity on host canine mitochondrial sequences, significantly increase the relative proportion of vector-borne bacterial DNA sequenced and increase the detection capability for elucidating infections by our NGS metabarcoding method. The increase in detection capability between our metabarcoding method with and without our blocking primer is particularly important as five more *E. canis* infections and three more *A. platys* infections were detected from canines when using the blocking primer in the first-step PCR. Accurate detection and characterisation of such pathogens is key when conducting a range of studies from large-scale surveillance investigations where reductions in sensitivity may impair the ability to provide apparent estimates that match the true prevalence of disease, through to clinical diagnostics on infirm canines where a missed infection may be fatal [7,9,43]. An increase in detected infections when using the blocking primer is similar to several studies which have reported exposition of previously occult DNA of interest using various blocking primers, in the context of both pathogen detection [26] and ancient DNA [30].

The number of detected infections by *M. haemocanis* was the same for samples regardless of whether the blocking primer was used. *M. haemocanis* infections were typically observed to have high numbers of reads, frequently over 10,000 per sample but reaching as high as 149,000 in one sample. This may result in *M. haemocanis* infections being easier to detect than other vector-borne bacterial species and thus minimising the possibility of missed infections even in the absence of blocking primers. 

Not only were more samples detected using our blocking primer but the relative proportion of canine vector-borne bacterial reads, relative to the total number of reads, was always higher when using this blocking primer. These results provide greater support for an improved metabarcoding sensitivity when utilising our blocking primer as if more pathogen 16S rDNA is amplified and sequenced relative to total amplified DNA, then the probability that infections with even low levels of circulating bacteria are detectable is also increased. Similar results were obtained by Tan and Liu (2018) [27] who were able to increase the relative abundance of desired protist reads when using metazoan-specific blocking primers in marine environments. 

Conversely, the relative proportion of canine mitochondrial sequences that were amplified by our bacterial primers was lower by an average of three times when using our blocking primer, a result that is further supported by the primer’s mean blocking efficiency of 25%. Not only do such results demonstrate the blocking primer’s ability to prevent amplifying primer cross-reactivity on host 12S rRNA sequences but they also signify an increase in relevant data obtainable when deep sequencing with our blocking primer. A typical MiSeq v3 chemistry run outputs between 3.3 to 15 Gb of data, [44] and if, as found in the current research, as much as 59% of the data output is the result of primer cross-reactivity on host (mtDNA), then a substantial cost is accrued in terms of labour, time and money to the researcher. Furthermore, if a large proportion of sampling effort is due to cross-reactive amplification then there is the potential for a significant masking effect whereby bacterial pathogen DNA may be missed as disproportional quantities of canine mtDNA are sequenced instead. 

The concentration of blocking primer used throughout this study was informed by prior experimentation elucidating a roughly 1:1 ratio of target to non-target DNA amplification. When this information was assessed, in conjunction with comparison from studies that had dealt with similar proportions of non-target DNA [26,27,45], a ratio of 3:1 blocking primer to amplifying primers was deemed sufficient. Experimentation using cPCR and evidence from a reduction of canine mitochondrial sequences demonstrates our chosen ratio to be effective, however, even with the addition of a blocking primer at this ratio there was still some continued amplification of canine mtDNA. On average 18% of each sample’s total reads was comprised of canine mitochondrial reads, with great variability on a sample by sample basis ranging from 0% to 64%. Taking this into consideration, an even higher ratio of blocking primer to amplifying primers may have reduced this cross-reactivity even more. Nonetheless, there is also a possibility that blocking primers could be generating off-target blocking effects on bacterial sequences, potentially reducing the detection of certain bacterial groups. For example, the 5′ end of our blocking primer has a run of at least seven bp that are capable of binding to bacterial 16S rRNA sequences, indicating there may be some potential for this blocking primer to anneal to bacterial sequences and reduce Wehi_Adp_515F efficiency. Therefore, it was important to keep blocking primer concentration as low as possible, whilst remaining effective at blocking desired targets [27,30]. 

### 3.2. DNA Extraction Kit Performance

Differences between results acquired throughout the range of parameters investigated were substantial between DNA extraction kit types, despite the same canine blood samples being used. Considering all kit performance results, the Qiagen DNeasy Blood and Tissue Kit performed the best within our NGS metabarcoding framework as it was able to detect the highest number of vector-borne bacterial infections (Table 4). This kit also elucidated a greater diversity of bacterial sequences, including some from putative pathogens, such as *Rickettsia* spp. and *Coxiella* spp., that were not found using the other extraction kits. Such results are of importance to our study given that the strength of our NGS-methodology is its ability to detect unusual or novel pathogens without *a priori* information regarding likely pathogens or their respective 16S rRNA sequences. Our findings, mirror those of other NGS kit comparison assessments that have found that DNA extraction kit employed can have a significant effect on the abundance and diversity of bacterial groups found [38,46]. For example, similar to our current results, Hart et al. (2015) found that a Qiagen spin-column based extraction method was better able to detect rare phyla when compared to four other extraction methods [46].

The considerably higher number of ASVs detected when using the QG kits was over 15 times the amount found by the automated WB and BC kits and almost five times that found by the BL kits, demonstrating the strength of the QG kits to liberate a higher diversity of bacterial sequences and allow for better blood microbiome characterisation (Table 3). Moreover, of all the kit types tested, the QG kit used the lowest quantity of initial starting material at just 100 µL of whole blood compared to 200 µL for both the BL and BC kits and 500 µL for the WB kit. Such findings provide weight to the argument that quantity of starting material alone does not necessarily improve sensitivity of pathogen detection and that DNA extraction methodology and chemistry may play a more significant role. This is further supported by the data on the number of raw and filtered reads obtained for the different kits, which found that the QG kits had less than that obtained for WB, BC and BL, an inevitable result of the samples from different kits having to be run on separate NGS flow cells to permit an adequate sampling depth (Table 3). Therefore, despite an initially smaller sample volume and a lower quantity of NGS read data, the superiority of the QG kit extraction process appears to have overcome such potential disadvantages and highlight the greatest number of infections and bacterial diversity of the four kits tested. 

A range of similar studies have explored the impacts of DNA extraction kits, either manual or automated, for pathogen detection using various downstream molecular methods, including cPCR, real-time PCR, restriction digest analysis and deep sequencing [34,35,36,46,47,48,49]. Overall, there are few consistent DNA extraction types that perform well in all contexts, with optimal extraction methods typically being very host, pathogen and sample-type specific [34,48]. For example, large differences in kit performance may be observed depending on whether the source material contains lots of PCR inhibitors, such as in faeces and blood, or on the biological properties of the pathogen of interest, e.g., viruses that are easy to lyse vs resilient spore-forming bacteria [34,36,38,49]. Moreover, the ability of different extraction kits to remove PCR-inhibitory molecules may play a large impact on obtainable downstream results. Blood contains potent PCR inhibitors such as haemoglobin that affects DNA polymerase activity and hence observed differences in kit performance could be due to how well different extraction processes remove such molecules [50]. 

In the current research, differences in extraction chemistry may be responsible for differences in bacterial pathogen detection between kits [35]. For example, neither of the automated WB and BC kits used a proteinase K digestion step, whilst both manual QG and BL kits did use this digestion process. Proteinase-based degradation may release DNA sequestered by peptides or from within bacterial cell walls, thus liberating more pathogen DNA, making it more readily available for PCR amplification [36]. Nonetheless, whilst both manual kits used proteinase K digestion only, the QG kit identified more infections than the automatic kits, meaning that this biochemical variable is likely just one of many responsible for differences in pathogen detection capability between extraction kits.

Depending on the extraction method utilised, the average DNA yield obtained also demonstrated marked differences. The automated BC and WB methodologies consistently extracted DNA yields in the range of hundreds of ng/µL with large variability between samples in the amount of DNA extracted (Table 3). In contrast, QG kits extracted an average of 13.9 ng/µL and BL kits extracted the lowest quantities at 3.4 ng/µL, with both spin-column based methods displaying much lower variability in nucleic acid yield. Such substantial differences in yield are due to the physical extraction methodology and chemistry employed by a given kit [48,51]. QG and BL kits are dependent on retention of DNA in a silica column matrix which becomes saturated, capping the maximum amount of DNA obtainable [46,48]. The Promega WB and BC automated methods use magnetic-bead based extraction whereby the large quantity of bead surface area available permits much larger amounts of DNA to be carried over between extraction stages [35,47]. Nonetheless, as previously explored, the higher DNA yields gained by the WB and BC kits did not necessarily translate to a greater number of vector-borne bacterial infections found. One explanation for this may be that the larger quantity of DNA extracted by the WB and BC kits meant total host DNA was in great excess, making efficient blocking by our *Canis*-mito-blk primer ineffective, resulting in bacterial infections being missed. 

With the advent of 16S rRNA bacterial metabarcoding, there has been a growing number of studies investigating the existence of endogenous bacterial contamination within DNA extraction kits and laboratory reagents [32,33,37]. Such findings underscore the importance of conducting no sample/reagent only negative controls within metabarcoding studies to correctly identify such contaminant species and thereby discern true species present within samples from those introduced by extraction kits [24,38,52,53]. In NGS studies that use a source material with a high density of bacteria, such as soil or faeces, this contamination typically has little effect as kit contaminant bacterial DNA is masked by that extracted from the source material [32,33]. However, when the source material has a low bacterial biomass, such as in blood, then kit contaminant bacterial DNA may be amplified and sequenced at similar levels to that of the source material, potentially competing and obscuring the microbiome signature of true bacterial inhabitants of the blood compartment [32,33]. An ever-growing list of common contaminant bacteria of kits highlights the tenacity of such organisms to survive and grow in a variety of environments, including ultra-pure water systems and other reagent manufacturing equipment and processes [32,37]. This creates a necessity to explore and identify differences in bacterial contamination between extraction kits which may influence the detection of true sample bacteria and demonstrate whether some kits are better suited to NGS applications.

In this study, bacterial taxa were considered an artificial kit contaminant if they had a read count of 100 or over in either of the two no sample/reagent only negative controls, extracted alongside the blood samples for each kit type. Large differences in the average percentage of all contaminant bacterial reads compared to total reads were observable between kit types with the automated BC (86%) and WB (80%) kits having on average over two times as much contamination as the QG kits and just under four times as much contamination as the BL kits (Figure 3). Such findings are supported by the DNA yield data found within extraction negative controls, from which WB and BC kits had the highest levels of negative control DNA, especially compared to the BL controls which had levels below the detection limit of the Qubit™ 3.0 fluorometer. Kit-derived bacterial contaminant DNA was dominated by just a few key genera in the WB (51% *Massilia* spp. and 28% *Pseudomonas* spp.) and BC kits (61% *Massilia* spp. and 24% *Cellulomonas* spp.) whilst the QG and BL kits demonstrated a larger diversity of kit contaminants, none of which comprised more than 9% of total reads (Supplementary Table S1). 

Disparate levels of kit contamination may be due to the physical properties of the extraction protocol used by each kit type. The automated WB and BC extraction methods utilise a series of seven wells all filled with between 700 to 1000 µL of lysis and wash buffers providing a large reagent pool from which contaminant bacteria could be introduced. In contrast, less than 600 µL of each reagent are added at any one time to the spin-column based QG and BL protocols with fewer wash steps from which endogenous bacteria could be added, lysed and their DNA extracted. The preponderance of a few key bacteria in the automated kit types may point to the growth or introduction of these species within the manufacturing process of these kits, whilst a larger diversity of less prevalent bacterial species in the QG and BL kits may be the product of small contamination events from the laboratory environment [32]. The large percentage of contaminant reads from the WB and BC kits may explain, in some part, the reduced number of vector-borne bacterial infections elucidated when compared to those found with the QG kits. Endogenous bacterial DNA may be concealing the detection of pathogen DNA when using these kits, particularly in blood samples with low levels of circulating pathogens [32,33]. 

An unexpected result identified by our assessment of DNA extraction kit performance was a significant difference in the amount of bacterial primer cross-reactivity on canine mitochondrial sequences between the kit types utilised. The BL kit obtained results that had a significantly higher amount of canine mitochondrial reads compared to the total, despite the use of the *Canis*-mito-blk primer, whilst the other three kit types had as low as 0.2% to 1.5% of total reads generated from cross-reactivity (Figure 4). An explanation may be the much lower DNA yields from the BL kits, that had, on average, just 3.4 ng/µL of DNA, a large proportion of which would be canine DNA. Such low sample yields may not have provided sufficient bacterial DNA template for 16S rRNA amplification, leading to more off-target amplification and sequencing of host mitochondrial sequences. Moreover, such low starting DNA yields may also be the reason for the poorer performance of the BL kits with respect to the number of vector-borne bacterial infections found, compared to the other kit types [51,54].

## 4. Conclusions

Taken together, the current study provides two complementary methodological amendments to improve the vector-borne bacterial detection capability of a previously developed metabarcoding protocol [22]. Firstly, employment of a 3′-spacer C3 blocking primer (*Canis*-mito-blk) to prevent bacterial amplification primers from cross-reacting on host mtDNA was demonstrated to improve the number of infections detected and the relative quantity of pathogen DNA relative to total DNA. Secondly, use of Qiagen’s DNeasy Blood and Tissue Kit appears superior to two automated methods utilising Promega’s Maxwell^®^ RSC 48 Instrument and Bioline’s manual ISOLATE II Genomic DNA Kit. This superiority was demonstrated via the DNeasy Blood and Tissue Kit’s ability to detect more infections as well as its lower quantities of kit contaminant bacterial DNA and generation of less off-target cross-reactivity on host mtDNA. Such methodological optimisation improves the power of our developed technique to unearth potentially latent vector-borne bacterial infections, a common phenomenon of many CVBDs where clinical signs may be followed by cyclical periods of remission when infections are hard to detect [7,55]. Furthermore, both amendments increased the diversity of different bacterial sequences elucidated, augmenting our protocol’s possibility of identifying rare or novel bacterial pathogens. To conclude, together these are key improvements to our metabarcoding method allowing it to mine and more fully characterise the range of bacterial pathogens in regions of the world where there is a plethora of different CVBDs alongside a dearth of applicable research [2,5,56]. 

## 5. Materials and Methods

### 5.1. Sampling and DNA Extraction

For blocking primer experiments, blood extracted DNA from 50 temple community dogs in Thailand were used as described in Huggins et al. (2019) [22] under Ethics Permit: OACKU-00758 and extracted using the E.Z.N.A.^®^ Blood DNA Mini Kit (Omega Biotek Inc., Norcross, GA, USA). For the comparison of DNA extraction kits in NGS metabarcoding applications, 50 whole blood samples were collected at four sites across Phnom Penh, Cambodia, from a mixture of pagoda temple communities, and locally owned and semi-domesticated dogs. Blood was only taken after obtaining informed consent from the relevant monk in the case of temple community dogs or the owner from local pet dogs. A qualified veterinarian conducted collection of two 1 mL blood samples per dog via cephalic puncture into anti-coagulation EDTA tubes on ice. These were then transferred to a −20 °C freezer upon return from the field. Approximately, 700 µL of blood from one sample, per dog, was then aliquoted to a separate Eppendorf tube and centrifuged at 12,000 rpm for 5 min in a portable LW-ZipCombo-C microcentrifuge (LW Scientific, Lawrenceville, GA, USA). From this, the blood plasma was disposed of and 250 µL of the Buffy Coat layer and surrounding erythrocytes was aliquoted and stored separately at −20 °C. Work in Cambodia was conducted under Ethics Permit: 1814620.1 from the University of Melbourne.

DNA extraction from the same 50 Cambodian canine whole blood or Buffy Coat samples was then carried out via one of four methods. Extractions were conducted according to manufacturer’s instructions in the same laboratory using the maximum possible starting quantity of sample for each kit. This was done to elucidate the pathogen detection capability and level of bacterial kit contaminants without subsequent researcher modifications to kit protocol and to assess kit utility for 16S rRNA metabarcoding as developed by the manufacturer. Extraction methods were as follows:

(1) Extraction of 500 µL of whole blood using the Maxwell^®^ RSC Whole Blood DNA Kit (Promega, Madison, WI, USA) on the automated Maxwell^®^ RSC 48 Instrument DNA extraction robot. This was conducted as per the manufacturer’s instructions and eluted in 100 µL of Ambion Nuclease-Free Water (Life Technologies, Carlsbad, CA, USA).
(2) Extraction of 250 µL of Buffy Coat layer using the Maxwell^®^ RSC Buffy Coat DNA Kit (Promega, Madison, WI, USA) on the Maxwell^®^ RSC 48 Instrument, conducted as per the manufacturer’s instructions and eluted in 100 µL of Ambion Nuclease-Free Water.
(3) Manual extraction of 200 µL of whole blood, using the ISOLATE II Genomic DNA Kit (Bioline, Memphis, TN, USA), using the manufacturer’s protocol with a final elution step in 50 µL.
(4) Manual extraction of 100 µL of whole blood, using the DNeasy Blood and Tissue Kit (Qiagen, Hilden, Germany) using the manufacturer’s protocol with a final elution step in 50 µL.

With all DNA extraction methods, two no blood/reagent only, DNA extraction negative controls were run per method to assess for levels of bacterial contamination in kits. Comparison of whole blood vs Buffy Coat based extraction was conducted due to a body of research suggesting that testing of specific blood fractions may improve sensitivity of detection for some blood-borne pathogens [40,41]. 

### 5.2. DNA Quantification

All DNA extractions were quantified on a Qubit™ 3.0 fluorometer (Life Technologies) using the Qubit™ dsDNA HS Assay Kit and means determined. Total DNA was also tested for in no blood/reagent only negative controls. 

### 5.3. Design of Blocking Primer

**Figure 5:**
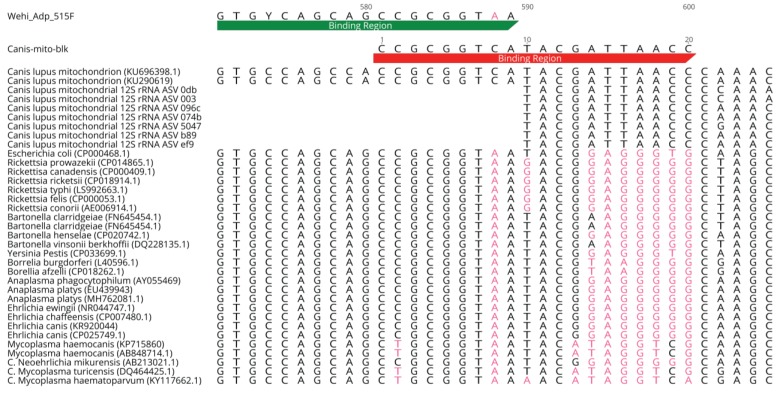
Alignment of pathogenic bacterial 16S rRNA sequences, canine 12S rRNA mitochondrial sequences, the forward bacterial primer Wehi_Adp_515F (green bar) and the *Canis*-mito-blk blocking primer (red bar) in the 5′ to 3′ direction. Nucleotide bases that differ from those at the relevant position in the *Canis*-mito-blk primer are coloured in light red, demonstrating dissimilarity between our designed blocking primer and bacterial 16S rRNA sequences but perfect complementarity with the 12S rRNA mitochondrial counterparts.

Previous work by Huggins et al. (2019) [22] testing a variety of bacteria-universal 16S rRNA targeting primer pairs at hypervariable regions V1 to V6 identified cross-reactivity on canine host DNA as a recurrent problem. Even the best performing primer pair; Wehi_Adp_515F (5′-GTGYCAGCAGCCGCGGTAA-3′) and Wehi_Adp_806R (5′-GGACTACNVGGGTATCTAAT-3′) still generated as many as 97% of total reads in some samples from primer cross-reactivity against canine 12S rRNA sequences, with an average of 47% across all samples found [22]. This was despite prior modifications to both primers to reduce degeneracy and thereby likelihood of cross-reactivity with canine 12S rRNA sequences [22]. Hence, to further prevent this, a *Canis lupus* 12S rRNA specific blocking primer was designed (*Canis*-mito-blk) with a 3′-spacer C3 to prevent DNA polymerase elongation of such mitochondrial sequences when used alongside the Wehi_Adp_515F and Wehi_Adp_806R, 16S rRNA primers. Blocking primer design was conducted in Geneious v. 11.1.5 (Biomatters Ltd.) using 26 16S rRNA sequences from pathogenically relevant bacterial species and an *Escherichia coli* reference sequence. Moreover, eight sequences of the *C. lupus* mitochondrial 12S rRNA gene (which cross-reacted with bacterial primers in prior experiments) were added, alongside the sequences for the bacterial 16S rRNA primers [22] (Figure 5). The 5′ end of our designed *Canis*-mito-blk primer has an 8 bp overlap with the bacterial forward primer Wehi_Adp_515F, then extending 11 bp downstream of this primer’s binding site, ending with a 3′ C3 spacer that prevents DNA polymerisation (Figure 1). The primer’s high complementarity to canine 12S rRNA sequences suggests it may block amplification of these sequences. Furthermore, base pair (bp) differences with bacterial 16S rRNA sequences prevent this blocking primer from efficiently binding to this region, allowing amplification of bacterial sequences to continue. The final design of *Canis*-mito-blk was 5′-CCGCGGTCATACGATTAACC/3SpC3/-3′. Figure 5 shows the location of the three bp differences between the Wehi_Adp_515F primer and the canine 12S rRNA mitochondrial sequences, where cross-reactivity can still occur.

### 5.4. Validation of Canis-mito-blk

To test for the specificity of *Canis*-mito-blk in vitro to canine 12S rRNA sequences, cPCR was conducted with and without the addition of the 3′ C3 spacer, plus a reverse canine 12S rRNA specific primer (5′-AATCCCAGTTTGGGTCTTAGC-3′) producing a 137 bp product. PCRs were 20 µL in total, comprising 10 µL of OneTaq^®^ 2X Master Mix with Standard Buffer (New England Biolabs, Ipswich, MA, USA) 0.4 μM of both forward and reverse 16S bacterial primers, 1.2 μM of both *Canis*-mito-blk and the 12S rRNA reverse primer, 1 µL of template DNA and Ambion Nuclease-Free Water. The ratio of blocking primer to bacterial primer should be informed by the ratio of cross-reactive 12S rRNA reads to 16S rRNA bacterial sequences [45], which is at a ratio of approximately 1:1 as calculated using prior data collected by Huggins et al. (2019) [22]. Therefore, to ensure blocking primer was in excess we used a 1:3 ratio of bacterial primer to blocking primer, as reported by similar studies, utilising blocking primers [26,27,29]. Thermocycling conditions were taken from Huggins et al. (2019) [22] and were 95 °C for 3 min, 35 cycles of 95 °C for 45 s, 56 °C for 60 s and 72 °C for 90 s with a final elongation at 72 °C for 10 min. Specific amplification of 12S rRNA sequences was conducted by visual observation of 137 bp bands on a 1.5% agarose gel using a GelDoc^TM^ XR + System (Bio-Rad, Hercules, CA, USA). PCR combinations using the blocking primer with and without bacterial 16S rRNA primers and the 3′ C3 spacer on DNA from non-infected and infected blood were conducted to ensure that *Canis*-mito-blk could amplify 12S rRNA sequences in the absence of the 3′ C3 spacer and completely block amplification when the 3′ C3 spacer was present.

### 5.5. Bacterial 16S rRNA Metabarcoding Assessment of Canis-mito-blk Efficacy

Fifty previously characterised blood-extracted DNA samples from Thai dogs [22] were deep-sequenced with and without the use of the *Canis*-mito-blk primer to assess its efficacy at preventing bacterial primer cross-reactivity and improving pathogen diversity characterisation. All first-step and second-step PCRs for deep-sequencing library preparation were conducted within a PCR hood after UV sterilisation. The first-step PCR consisted of 10 µL of OneTaq^®^ 2X Master Mix, 0.4 μM of both forward and reverse 16S bacterial primers with NGS overhang sequences, 1.2 µL of *Canis*-mito-blk (for the 50 samples using the blocking primer), 1 µL of template DNA with the whole reaction made up to a total volume of 20 µL with Ambion Nuclease-Free Water. Bacteria primer sequences with the addition of NGS overhangs (underlined) were WehiNGS_Adp_F (5′-GTGACCTATGAACTCAGGAGTCGTGYCAGCAGCCGCGGTAA-3′) and WehiNGS_Adp_R (5′-CTGAGACTTGCACATCGCAGCGGACTACNVGGGTATCTAAT-3′). The thermocycling profile was used with just 20 amplification cycles, instead of 35. PCR product was then cleaned using 1X Ampure Beads (Beckman Coulter, Brea, CA, USA) and checked for quality on an Agilent 2200 Tape Station (Agilent Technologies, Santa Clara, CA, USA) before proceeding. Two no-template PCR negative controls and four uniquely identifiable positive controls were also included in this first-step PCR. Due to the need to assess for possible cross-contamination between samples during deep-sequencing library preparation or Illumina indexing errors, artificial positive control constructs were designed and synthesised as recommended by Kim et al. (2017) [24]. Positive controls were a gBlock synthetic DNA construct (Integrated DNA Technologies, Coralville, IA, USA) comprised of a uniquely identifiable 253 bp 16S rRNA sequence from *Aliivibrio fischeri*, a bacterial endosymbiont found within the Bobtail squid, *Euprymna scolopes* and its environment [57,58]. The *A. fischeri* sequence is flanked by the relevant 16S rRNA primer binding sites to allow for positive control amplification and is 253 bp long to match the typical length of fragments amplified by the 16S rRNA primers on naturally occurring bacterial species. This bacterial sequence was chosen as there is very little possibility that *A. fischeri* would be found as an environmental or laboratory contaminant, nor would it be found in canine blood, making it uniquely identifiable. Supplementary file 2 has the sequence of this positive control construct. The second-step PCR for addition of library indices and deep sequencing was then conducted according to Huggins et al. (2019) [22] using an Illumina MiSeq (Illumina, San Diego, CA, USA) with paired-end 600-cycle v3 chemistry at the Walter and Eliza Hall Institute (WEHI) Proteomics Facility, Parkville, Australia. Paired-end sequencing was conducted to assure read quality as only reads that had identical overlapping R1 and R2 regions were passed on to downstream analysis. 

### 5.6. Bacterial 16S rRNA Metabarcoding Comparison of DNA Extraction Kit Performance

The 50 Cambodian dog blood samples and two reagent-only extractions per method were conducted using the previously described protocols on the Maxwell^®^ RSC Whole Blood DNA Kit (WB), the Maxwell^®^ RSC Buffy Coat DNA Kit (BC), the Bioline ISOLATE II Genomic DNA Kit (BL) and the Qiagen DNeasy Blood and Tissue Kit (QG). Samples were then prepared for deep-sequencing in the same manner as those used in the blocking primer comparison, with 112 samples indexed and multiplexed per flow cell. Each batch of 50 DNA extractions per kit included an additional seven controls that were deep sequenced. These were; two reagent-only DNA extraction negative controls, two no-template PCR negative controls, two uniquely identifiable *A. fischeri* positive controls and one ZymoBIOMICS Microbial Community DNA Standard (Zymo Research, Irvine, CA, USA) positive control to assess for PCR amplification bias.

### 5.7. Bioinformatics

Raw NGS data was demultiplexed using in-house software at WEHI and then imported into the QIIME 2 (v. 2019.1) environment for bioinformatic analysis [59]. Raw data were processed according to Huggins et al. (2019) [22] with the only adaptation being that amplicon sequence variants (ASVs) were generated and taxonomically assigned as opposed to sequence clustering and formation of operational taxonomic units (OTUs), reflecting recent research into microbiome analysis best practice [60,61]. Alpha rarefaction plots were generated, using the functions MAFFT [62] and FastTree 2 [63] to ensure that OTU diversity plateaued and hence a sufficient sequencing depth had been achieved. All NGS data generated exploring the use of our blocking primer is available from the NCBI BioProject database, with BioProject ID: PRJNA528154 and SRA data accession numbers SRR10895013 to SRR10895109. All data that was obtained to compare the performance of DNA extraction kits is available under BioProject ID: PRJNA601241 and SRA data accession numbers SRR10959940 to SRR10960037 (WB and BC), SRR10972728 to SRR10972776 (BL) and SRR10972824 to SRR10972872 (QG).

The read threshold for a true infection by a bacterial pathogen was determined as the mean reads of non-control samples that were identified as having sequences from *A. fischeri* (the positive control construct) within them. The appearance of these positive control sequences in other samples could be due to occasional index misreading or hybridisation errors during Illumina sequencing as well as low-level cross-contamination during library preparation [24]. Read threshold values were never higher than 62 reads, which was the NGS run found to have the highest amount of cross-contamination. Appearance of contaminant sequences across the 96-well plate was scattered and not concentrated near the positive control samples, implying that library preparation was unlikely to be the major cause of such cross-contamination. 

Taxa found in no-reagent DNA extraction negative controls and no-template PCR negative controls were deducted from the overall dataset used for comparison of pathogen detection capability. This negative control data was used for comparison of contaminant bacterial DNA arising from extraction kits. 

The results from the ZymoBIOMICS Microbial Community DNA Standards were compared to the expected read composition. A high level of similarity was found, demonstrating no PCR amplification bias by our chosen bacterial 16S rRNA primers. 

### 5.8. Data Analysis of Blocking Primer Performance

Data analysis to calculate blocking primer performance, including biodiversity index calculations and blocking efficiency was conducted in Excel 2016 v. 1803 (Microsoft, Redmond, WA, USA). Statistical analyses were carried out using Minitab v. 19.2 (Minitab LLC, State College, PA, USA). 

The percentage of relevant vector-borne bacterial reads (*A. platys*, *E. canis* and *Mycoplasma haemocanis*) compared to the total reads for each sample was calculated, to account for differences in sampling effort between samples, observable as differing numbers of total sample reads. Data normality was tested for using the Anderson–Darling test. The mean percentage of bacterial reads compared to total reads, standard error and Wilcoxon paired t-test statistics were calculated to determine if differences in means between paired samples, i.e., the same sample with and without the blocking primer, were statistically significant. Results were interpreted as significant at the *p* < 0.01 level. The same analysis was carried out to elucidate the average proportion of canine mitochondrial cross-reactivity between the same samples with and without the blocking primer. In addition, blocking primer efficiency was calculated as defined by Tan and Liu (2018) [27] in the equation (X−Y) ÷Y. Here, X is defined as the ratio of mitochondrial cross-reactive reads to the total of all bacterial reads without the blocking primer for a sample and Y is the ratio of mitochondrial reads to total bacterial reads with the blocking primer for each sample. This equation works on the assumptions that *Canis*-mito-blk is specific to canine mitochondrial sequences and, therefore, that non-mitochondrial sequences will not be inhibited. Following this, the mean and standard error of all sample blocking efficiencies was calculated to get an approximation of overall *Canis*-mito-blk blocking efficiency. 

Read cut-off values to ascertain if a sample had a particular vector-borne bacterial species present, i.e., was a true infection were defined according to Huggins et al. (2019) [22]. In brief, the mean number of unique *A. fischeri* positive control construct reads that appeared in non-positive control samples was calculated across all samples, with and without the blocking primer. The means of these reads was taken to demonstrate the average quantity of Illumina indexing errors or potential cross-contamination between samples during library preparation. Therefore, read values under these means were not counted as true infections while those above were counted. The total number of infections was tallied across the same 50 samples with and without the blocking primer to assess sensitivity of pathogen detection. 

Biodiversity indices were calculated for all bacterial reads across all samples with and without the blocking primer to elucidate whether their addition improved the ability of our methodology to characterise bacterial species diversity and, therefore, the potential to detect rare or novel species. We used two biodiversity indices; Shannon–Wiener (H) and Simpson’s (D) index, which both account for species richness (number of bacterial species) and species abundance (approximated via number of reads of each species) [64,65,66,67,68]. 

### 5.9. Data Analysis of DNA Extraction Kit Performance

DNA extraction kit performance was assessed using 43 blood samples that were successfully deep-sequenced from all four kit types used. DNA yield, number of vector-borne bacterial infections detected, mean percentages of contaminant bacterial reads and mean percentage of bacterial primer cross-reactivity on canine mitochondrial sequences, relative to total reads were all calculated. Statistical analysis to compare mean percentages of cross-reactivity and mean percentages of bacterial contaminant sequences were calculated using Minitab v. 19.2 using one-way ANOVAs and Tukey pairwise comparisons to assess which means were statistically significant between kit types at the *p* < 0.05 level. 

Regarding the assessment of levels of bacterial contamination from kits, i.e., artificial contamination, a bacterial taxon was deemed to be an artificial contaminant if it had a read count of ≥100 in either of the no blood/reagent only DNA extraction negative controls, run for that kit type. For every sample, the number of reads for all artificial kit contaminant taxa was totalled and a percentage calculated relative to the total number of reads. Artificial kit contaminants are different from natural contaminants, such as bacteria residing on canine skin, that may be picked up during sampling via venepuncture or the sampling environment, these naturally derived contaminants were not assessed. 

## Figures and Tables

**Table 1:** Comparison of total number of infections detected when *Canis*-mito-blk was absent or present. Infections were deemed true when the total number of reads of a particular pathogen were over the read cut-off value as defined in the Materials and Methods.

Vector-Borne Bacteria	Total Infections: Blocking Primer Absent	Total Infections: Blocking Primer Present
*A. platys*	17	20
*E. canis*	19	24
*M. haemocanis*	19	19

**Table 2:** Comparison of biodiversity indices when blocking primers (BP) were absent or present. The higher the index, the greater the relative diversity.

Bacterial Diversity: Shannon–Wiener (H) Index		Bacterial Diversity: Simpson (D) Index	
BP Absent	BP Present	BP Absent	BP Present
1.81	2.3	3.15	4.07

**Table 3:** Comparison of mean DNA quantity and standard error (S.E.) from the same blood samples extracted with different kits and comparison of contaminant DNA in kit reagents alone. Includes post-bioinformatic analysis results of total raw and filtered reads accrued between different kit types as well as total Amplicon Sequence Variants (ASVs), i.e., diversity of DNA sequences found.

DNA Extraction Kit	Mean DNA Quantity ± S.E. (ng/µL)	DNA Quantity in ExtractionNegative Controls (ng/µL)	Total Raw Reads	Reads Post-Filtering	Total ASVs
Promega Whole Blood (WB)	483.8 (±150.9)	0.005–0.017	5,351,987	3,857,213	435
Promega Buffy Coat (BC)	680.2 (±394.7)	0.012–0.024	5,916,458	4,716,590	428
Bioline Whole Blood (BL)	3.4 (±0.6)	<0.001	5,935,103	4,188,285	1401
Qiagen Whole Blood (QG)	13.9 (±1.1)	0.001–0.014	4,462,788	3,559,370	6683

**Table 4:** Comparison of total number of infections detected of the three main bacterial pathogens by different extraction kits. Infections were deemed true when the total number of reads of a pathogen were over the read cut-off value as defined in the Materials and Methods.

Vector-Borne Bacteria	Promega Whole Blood (WB)	Promega Buffy Coat (BC)	Bioline Whole Blood (BL)	Qiagen Whole Blood (QG)
*A. platys*	18	17	13	22
*E. canis*	7	7	6	8
*M. haemocanis*	3	4	4	5

## Supplementary Materials

The following are available online at https://www.mdpi.com/2076-0817/9/4/258/s1, Table S1: Most abundant bacterial ASVs found within four different DNA extraction kits’ reagents. Supplementary file 2: Sequence of our unique positive control gBlock DNA construct.

## References

[1] Maggi R.G., Krämer F. (2019). A review on the occurrence of companion vector-borne diseases in pet animals in Latin America. Parasit. Vectors.

[2] Traub R.J., Irwin P., Dantas-Torres F., Tort G.P., Labarthe N.V., Inpankaew T., Gatne M., Linh B.K., Schwan V., Watanabe M. (2015). Toward the formation of a Companion Animal Parasite Council for the Tropics (CAPCT). Parasit. Vectors.

[3] Koh F.X., Panchadcharam C., Tay S.T. (2016). Vector-borne diseases in stray dogs in peninsular Malaysia and molecular detection of *Anaplasma* and *Ehrlichia* spp. from *Rhipicephalus sanguineus* (Acari: Ixodidae) ticks. J. Med. Entomol..

[4] Rucksaken R., Maneeruttanarungroj C., Maswanna T., Sussadee M., Kanbutra P. (2019). Comparison of conventional polymerase chain reaction and routine blood smear for the detection of *Babesia canis*, *Hepatozoon canis*, *Ehrlichia canis*, and *Anaplasma platys* in Buriram Province, Thailand. Vet. World.

[5] Irwin P.J., Jefferies R. (2004). Arthropod-transmitted diseases of companion animals in Southeast Asia. Trends Parasitol..

[6] Inpankaew T., Hii S.F., Chimnoi W., Traub R.J. (2016). Canine vector-borne pathogens in semi-domesticated dogs residing in northern Cambodia. Parasit. Vectors.

[7] Mylonakis M.E., Harrus S., Breitschwerdt E.B. (2019). An update on the treatment of canine monocytic ehrlichiosis (*Ehrlichia canis*). Vet. J..

[8] Kaewmongkol G., Lukkana N., Yangtara S., Kaewmongkol S., Thengchaisri N., Sirinarumitr T., Jittapalapong S., Fenwick S.G. (2017). Association of *Ehrlichia canis*, Hemotropic *Mycoplasma* spp. and *Anaplasma platys* and severe anemia in dogs in Thailand. Vet. Microbiol..

[9] Little S.E. (2010). Ehrlichiosis and anaplasmosis in dogs and cats. Vet. Clin. North Am. Small Anim. Pract..

[10] Carrade D.D., Foley J.E., Borjesson D.L., Sykes J.E. (2009). Canine granulocytic anaplasmosis: A review. J. Vet. Intern. Med..

[11] Chomel B.B., Mac Donald K.A., Kasten R.W., Chang C.C., Wey A.C., Foley J.E., Thomas W.P., Kittleson M.D. (2001). Aortic valve endocarditis in a dog due to *Bartonella clarridgeiae*. J. Clin. Microbiol..

[12] Hii S.F., Kopp S.R., Abdad M.Y., Thompson M.F., O’Leary C.A., Rees R.L., Traub R.J. (2011). Molecular evidence supports the role of dogs as potential reservoirs for *Rickettsia felis*. Vector-Borne Zoonotic Dis..

[13] Ng-Nguyen D., Hii S.-F., Hoang M.-T.T., Nguyen V.-A.T., Rees R., Stenos J., Traub R.J. (2020). Domestic dogs (*Canis familiaris*) are natural mammalian reservoirs for flea-borne spotted fever caused by *Rickettsia felis*. Sci. Rep..

[14] Fourie J.J., Evans A., Labuschagne M., Crafford D., Madder M., Pollmeier M., Schunack B. (2019). Transmission of *Anaplasma phagocytophilum* (Foggie, 1949) by *Ixodes ricinus* (Linnaeus, 1758) ticks feeding on dogs and artificial membranes. Parasit. Vectors.

[15] Krämer F., Hüsken R., Krüdewagen E.M., Deuster K., Blagburn B., Straubinger R.K., Butler J., Fingerle V., Charles S., Settje T. (2019). Prevention of transmission of *Borrelia burgdorferi* sensu lato and *Anaplasma phagocytophilum* by *Ixodes* spp. ticks to dogs treated with the Seresto^®^ collar (imidacloprid 10% + flumethrin 4.5%). Parasitol. Res..

[16] Krupka I., Straubinger R.K. (2010). Lyme Borreliosis in dogs and cats: Background, diagnosis, treatment and prevention of infections with *Borrelia burgdorferi* sensu stricto. Vet. Clin. North Am. Small Anim. Pract..

[17] Bakken J.S., Dumler J.S. (2015). Human granulocytic anaplasmosis. Infect. Dis. Clin. North Am..

[18] Takhampunya R., Korkusol A., Pongpichit C., Yodin K., Rungrojn A., Chanarat N., Promsathaporn S., Monkanna T., Thaloengsok S., Tippayachai B. (2019). Metagenomic approach to characterizing disease epidemiology in a disease-endemic environment in northern Thailand. Front. Microbiol..

[19] Vayssier-Taussat M., Moutailler S., Michelet L., Devillers E., Bonnet S., Cheval J., Hébert C., Eloit M. (2013). Next generation sequencing uncovers unexpected bacterial pathogens in ticks in western Europe. PLoS ONE.

[20] Greay T.L., Gofton A.W., Paparini A., Ryan U.M., Oskam C.L., Irwin P.J. (2018). Recent insights into the tick microbiome gained through next-generation sequencing. Parasit. Vectors.

[21] Huggins L.G., Koehler A.V., Ng-Nguyen D., Wilcox S., Schunack B., Inpankaew T., Traub R.J. (2019). A novel metabarcoding diagnostic tool to explore protozoan haemoparasite diversity in mammals: A proof-of-concept study using canines from the tropics. Sci. Rep..

[22] Huggins L.G., Koehler A.V., Ng-Nguyen D., Wilcox S., Schunack B., Inpankaew T., Traub R.J. (2019). Assessment of a metabarcoding approach for the characterisation of vector-borne bacteria in canines from Bangkok, Thailand. Parasit. Vectors.

[23] Liu M., Ruttayaporn N., Saechan V., Jirapattharasate C., Vudriko P., Moumouni P.F.A., Cao S., Inpankaew T., Ybañez A.P., Suzuki H. (2016). Molecular survey of canine vector-borne diseases in stray dogs in Thailand. Parasitol. Int..

[24] Kim D., Hofstaedter C.E., Zhao C., Mattei L., Tanes C., Clarke E., Lauder A., Sherrill-Mix S., Chehoud C., Kelsen J. (2017). Optimizing methods and dodging pitfalls in microbiome research. Microbiome.

[25] Taanman J.W. (1999). The mitochondrial genome: Structure, transcription, translation and replication. Biochim. Biophys. Acta-Bioenerg..

[26] Gofton A.W., Oskam C.L., Lo N., Beninati T., Wei H., McCarl V., Murray D.C., Paparini A., Greay T.L., Holmes A.J. (2015). Inhibition of the endosymbiont “*Candidatus* Midichloria mitochondrii” during 16S rRNA gene profiling reveals potential pathogens in *Ixodes* ticks from Australia. Parasit. Vectors.

[27] Tan S., Liu H. (2018). Unravel the hidden protistan diversity: Application of blocking primers to suppress PCR amplification of metazoan DNA. Appl. Microbiol. Biotechnol..

[28] Vestheim H., Jarman S.N. (2008). Blocking primers to enhance PCR amplification of rare sequences in mixed samples – a case study on prey DNA in Antarctic krill stomachs. Front. Zool..

[29] Leray M., Agudelo N., Mills S.C., Meyer C.P. (2013). Effectiveness of annealing blocking primers versus restriction enzymes for characterization of generalist diets: Unexpected prey revealed in the gut contents of two coral reef fish species. PLoS ONE.

[30] Boessenkool S., Epp L.S., Haile J., Bellemain E., Edwards M., Coissac E., Willerslev E., Brochmann C. (2012). Blocking human contaminant DNA during PCR allows amplification of rare mammal species from sedimentary ancient DNA. Mol. Ecol..

[31] Hino A., Maruyama H., Kikuchi T. (2016). A novel method to assess the biodiversity of parasites using 18S rDNA Illumina sequencing; parasitome analysis method. Parasitol. Int..

[32] Eisenhofer R., Minich J.J., Marotz C., Cooper A., Knight R., Weyrich L.S. (2019). Contamination in low microbial biomass microbiome studies: Issues and recommendations. Trends Microbiol..

[33] Glassing A., Dowd S.E., Galandiuk S., Davis B., Chiodini R.J. (2016). Inherent bacterial DNA contamination of extraction and sequencing reagents may affect interpretation of microbiota in low bacterial biomass samples. Gut Pathog..

[34] Podnecky N.L., Elrod M.G., Newton B.R., Dauphin L.A., Shi J., Chawalchitiporn S., Baggett H.C., Hoffmaster A.R., Gee J.E. (2013). Comparison of DNA extraction kits for detection of *Burkholderia pseudomallei* in spiked human whole blood using real-time PCR. PLoS ONE.

[35] Yang G., Erdman D.E., Kodani M., Kools J., Bowen M.D., Fields B.S. (2011). Comparison of commercial systems for extraction of nucleic acids from DNA/RNA respiratory pathogens. J. Virol. Methods.

[36] Riemann K., Adamzik M., Frauenrath S., Egensperger R., Schmid K.W., Brockmeyer N.H., Siffert W. (2007). Comparison of manual and automated nucleic acid extraction from whole-blood samples. J. Clin. Lab. Anal..

[37] Laurence M., Hatzis C., Brash D.E. (2014). Common contaminants in next-generation sequencing that hinder discovery of low-abundance microbes. PLoS ONE.

[38] Velásquez-Mejía E.P., de la Cuesta-Zuluaga J., Escobar J.S. (2018). Impact of DNA extraction, sample dilution, and reagent contamination on 16S rRNA gene sequencing of human feces. Appl. Microbiol. Biotechnol..

[39] Werren J.H., Baldo L., Clark M.E. (2008). *Wolbachia*: Master manipulators of invertebrate biology. Nat. Rev. Microbiol..

[40] Alexandre N., Santos A.S., Núncio M.S., de Sousa R., Boinas F., Bacellar F. (2009). Detection of *Ehrlichia canis* by polymerase chain reaction in dogs from Portugal. Vet. J..

[41] Mylonakis M.E., Koutinas A.F., Billinis C., Leontides L.S., Kontos V., Papadopoulos O., Rallis T., Fytianou A. (2003). Evaluation of cytology in the diagnosis of acute canine monocytic ehrlichiosis (*Ehrlichia canis*): A comparison between five methods. Vet. Microbiol..

[42] Morris E.K., Caruso T., Buscot F., Fischer M., Hancock C., Maier T.S., Meiners T., Müller C., Obermaier E., Prati D. (2014). Choosing and using diversity indices: Insights for ecological applications from the German Biodiversity Exploratories. Ecol. Evol..

[43] Greiner M., Gardner I.A. (2000). Epidemiologic issues in the validation of veterinary diagnostic tests. Prev. Vet. Med..

[44] Ravi R.K., Walton K., Khosroheidari M. (2018). Miseq: A next generation sequencing platform for genomic analysis. Methods in Molecular Biology.

[45] Vestheim H., Deagle B.E., Jarman S.N. (2010). Application of blocking oligonucleotides to improve signal-to-noise ratio in a PCR. Methods in Molecular Biology.

[46] Hart M.L., Meyer A., Johnson P.J., Ericsson A.C. (2015). Comparative evaluation of DNA extraction methods from feces of multiple host species for downstream next-generation sequencing. PLoS ONE.

[47] Wiesinger-Mayr H., Jordana-Lluch E., Martró E., Schoenthaler S., Noehammer C. (2011). Establishment of a semi-automated pathogen DNA isolation from whole blood and comparison with commercially available kits. J. Microbiol. Methods.

[48] Schiebelhut L.M., Abboud S.S., Gómez Daglio L.E., Swift H.F., Dawson M.N. (2017). A comparison of DNA extraction methods for high-throughput DNA analyses. Mol. Ecol. Resour..

[49] Phillips K., McCallum N., Welch L. (2012). A comparison of methods for forensic DNA extraction: Chelex-100^®^ and the QIAGEN DNA Investigator Kit (manual and automated). Forensic Sci. Int. Genet..

[50] Sidstedt M., Hedman J., Romsos E.L., Waitara L., Wadsö L., Steffen C.R., Vallone P.M., Rådström P. (2018). Inhibition mechanisms of hemoglobin, immunoglobulin G, and whole blood in digital and real-time PCR. Anal. Bioanal. Chem..

[51] Hall C.M., Jaramillo S., Jimenez R., Stone N.E., Centner H., Busch J.D., Bratsch N., Roe C.C., Gee J.E., Hoffmaster A.R. (2019). *Burkholderia pseudomallei*, the causative agent of melioidosis, is rare but ecologically established and widely dispersed in the environment in Puerto Rico. PLoS Negl. Trop. Dis..

[52] Zinger L., Bonin A., Alsos I.G., Bálint M., Bik H., Boyer F., Chariton A.A., Creer S., Coissac E., Deagle B.E. (2019). DNA metabarcoding—Need for robust experimental designs to draw sound ecological conclusions. Mol. Ecol..

[53] Tedersoo L., Drenkhan R., Anslan S., Morales-Rodriguez C., Cleary M. (2019). High-throughput identification and diagnostics of pathogens and pests: Overview and practical recommendations. Mol. Ecol. Resour..

[54] Vijayvargiya P., Jeraldo P.R., Thoendel M.J., Greenwood-Quaintance K.E., Esquer Garrigos Z., Sohail M.R., Chia N., Pritt B.S., Patel R. (2019). Application of metagenomic shotgun sequencing to detect vector-borne pathogens in clinical blood samples. PLoS ONE.

[55] Beck A., Huber D., Antolić M., Anzulović Ž., Reil I., Polkinghorne A., Baneth G., Beck R. (2019). Retrospective study of canine infectious haemolytic anaemia cases reveals the importance of molecular investigation in accurate postmortal diagnostic protocols. Comp. Immunol. Microbiol. Infect. Dis..

[56] Colella V., Nguyen V.L., Tan D.Y., Lu N., Fang F., Zhijuan Y., Wang J., Liu X., Chen X., Dong J. (2020). Zoonotic vectorborne pathogens and ectoparasites of dogs and cats in Asia. Emerg. Infect. Dis..

[57] Ruby E.G., Urbanowski M., Campbell J., Dunn A., Faini M., Gunsalus R., Lostroh P., Lupp C., McCann J., Millikan D. (2005). Complete genome sequence of *Vibrio fischeri*: A symbiotic bacterium with pathogenic congeners. Proc. Natl. Acad. Sci. USA.

[58] Nyholm S.V., McFall-Ngai M.J. (2004). The winnowing: Establishing the squid-*Vibrio* symbiosis. Nat. Rev. Microbiol..

[59] Bolyen E., Rideout J.R., Dillon M.R., Bokulich N.A., Abnet C.C., Al-Ghalith G.A., Alexander H., Alm E.J., Arumugam M., Asnicar F. (2019). Reproducible, interactive, scalable and extensible microbiome data science using QIIME 2. Nat. Biotechnol..

[60] Langille M.G.I., Nearing J.T., Douglas G.M., Comeau A.M. (2018). Denoising the Denoisers: An independent evaluation of microbiome sequence error-correction approaches. PeerJ.

[61] Knight R., Vrbanac A., Taylor B.C., Aksenov A., Callewaert C., Debelius J., Gonzalez A., Kosciolek T., McCall L.I., McDonald D. (2018). Best practices for analysing microbiomes. Nat. Rev. Microbiol..

[62] Katoh K., Standley D.M. (2013). MAFFT multiple sequence alignment software version 7: Improvements in performance and usability. Mol. Biol. Evol..

[63] Price M.N., Dehal P.S., Arkin A.P. (2010). FastTree 2—Approximately maximum-likelihood trees for large alignments. PLoS ONE.

[64] Spellerberg I.F., Fedor P.J. (2003). A tribute to Claude-Shannon (1916–2001) and a plea for more rigorous use of species richness, species diversity and the “Shannon-Wiener” Index. Glob. Ecol. Biogeogr..

[65] Gill C.A., Joanes D.N. (1979). Bayesian estimation of Shannon’s index of diversity. Biometrika.

[66] Hunter P.R., Gaston M.A. (1988). Numerical index of the discriminatory ability of typing systems: An application of Simpson’s index of diversity. J. Clin. Microbiol..

[67] Ogedengbe M.E., El-Sherry S., Ogedengbe J.D., Chapman H.D., Barta J.R. (2018). Phylogenies based on combined mitochondrial and nuclear sequences conflict with morphologically defined genera in the eimeriid coccidia (Apicomplexa). Int. J. Parasitol..

[68] Allaby M. (2010). A Dictionary of Ecology.

