# Supplementary material for: A Host-Specific Blocking Primer Combined with Optimal DNA Extraction Improves the Detection Capability of a Metabarcoding Protocol for Canine Vector-Borne Bacteria
Source: Pathogens. 2020 Apr 1;9(4):258. doi: 10.3390/pathogens9040258 (PMC7238069; doi:10.3390/pathogens9040258)
Supplement: Supplementary file 1 [file pathogens-09-00258-s001.zip › pathogens-757433-supplementary/Supplementary File 1 for BP - Contam Bacteria.docx]

**Supplementary File 1 (.docx).**

**Table S1. Most abundant bacterial ASVs found within four different DNA extraction kits’ reagents.** Only bacterial ASVs that comprise over 0.1% of total reads for the respective extraction kit’s NGS run and that were able to be classified below the rank of class are included. Maxwell® RSC Whole Blood DNA Kit = WB, Maxwell® RSC Buffy Coat DNA Kit = BC, Bioline ISOLATE II Genomic DNA Kit = BL, Qiagen DNeasy Blood & Tissue Kit = QG.

| **WB Kit Contaminant** | **Reads (% of Total)** |
| --- | --- |
| *Massilia* spp. | 51.36 |
| *Pseudomonas* spp. | 28.16 |
| *Leifsonia* spp. | 0.17 |
| **BC Kit Contaminant** | **Reads (% of Total)** |
| *Massilia* spp. | 61.20 |
| *Cellulomonas* spp. | 24.33 |
| *Leifsonia* spp. | 0.36 |
| **BL Kit Contaminant** | **Reads (% of Total)** |
| Burkholderiaceae | 4.79 |
| *Pseudomonas* spp. | 4.66 |
| *Acinetobacter* spp. | 1.02 |
| *Janthinobacterium* spp. | 0.82 |
| *Sphingomonas* spp. | 0.67 |
| Enterobacteriaceae | 0.61 |
| *Massilia* spp. | 0.36 |
| *Aquabacterium* spp. | 0.26 |
| *Pedobacter* spp. | 0.25 |
| *Undibacterium* spp. | 0.15 |
| **QG Kit Contaminant** | **Reads (% of Total)** |
| Chloroplast | 7.95 |
| Burkholderiaceae | 4.33 |
| *Parcubacteria* spp. | 3.38 |
| Mitochondria | 1.63 |
| *Prevotella* spp. | 1.42 |
| Candidatus *Nomurabacteria* | 1.24 |
| *Flavobacterium* spp. | 1.20 |
| *Acinetobacter* spp. | 1.13 |
| *Fodinicola* spp. | 1.08 |
| Saccharimonadales | 1.03 |
| *Legionella* spp. | 0.77 |
| *Sphingomonas* spp. | 0.62 |
| *Aquicella* spp. | 0.41 |
| *Gracilibacteria* spp. | 0.34 |
| Candidatus *Zambryskibacteria* | 0.27 |
| Candidatus *Magasanikbacteria* | 0.25 |
| *Oligoflexales* spp. | 0.25 |
| Saprospiraceae | 0.24 |
| Sphingomonadaceae | 0.24 |
| Lachnospiraceae | 0.22 |
| *Methylobacterium* spp. | 0.17 |
| *Faecalibacterium* spp. | 0.16 |
| *Polynucleobacter* spp. | 0.15 |
| Weeksellaceae | 0.13 |
| Babeliales | 0.12 |
| *Arcicella* spp. | 0.11 |
