# Supplementary material for: A Host-Specific Blocking Primer Combined with Optimal DNA Extraction Improves the Detection Capability of a Metabarcoding Protocol for Canine Vector-Borne Bacteria
Source: Pathogens. 2020 Apr 1;9(4):258. doi: 10.3390/pathogens9040258 (PMC7238069; doi:10.3390/pathogens9040258)
Supplement: Supplementary file 1 [file pathogens-09-00258-s001.zip › pathogens-757433-supplementary/Supplementary File 2 for blocking primer.docx]

**Supplementary File 2 (.docx). Sequence of our unique positive control gBlock DNA construct.** Positive control sequence is comprised of WehiNGS_Adp primer binding sites (underlined) and the V4 region of the *Aliivibrio fischeri* 16S rRNA gene.

5’- GTGCCAGCAGCCGCGGTAATACGGAGGGTGCGAGCGTTAATCGGAATTACTGGGCGTAAAGCGCATGCAGGTGGTTCATTAAGTCAGATGTGAAAGCCCGGGGCTCAACCTCGGAACCGCATTTGAAACTGGTGAACTAGAGTGCTGTAGAGGGGGGTAGAATTTCAGGTGTAGCGGTGAAATGCGTAGAGATCTGAAGGAATACCAGTGGCGAAGGCGGCCCCCTGGACAGACACTGACACTCAGATGCGAAAGCGTGGGGAGCAAACAGGATTAGATACCCTGGTAGTCC - 3’
